# Pattern recognition receptors and their roles in antiviral innate immunity in livestock

**DOI:** 10.3389/fimmu.2025.1746193

**Published:** 2026-01-28

**Authors:** Shujing Liu, Junpeng Qi, Yuxin Wang, Minglu Liu, Monong Su, Liang Wang, Minghua Li, Zimo Zhao, Jilin Chen

**Affiliations:** 1 Department of Nephrology, The First Affiliated Hospital of Dalian Medical University, Dalian, Liaoning, China; 2 Research and Teaching Department of Comparative Medicine, Dalian Medical University, Dalian, Liaoning, China; 3 Kunming Institute of Zoology, Chinese Academy of Sciences, Kunming, Yunnan, China; 4 School of Biological Sciences, University of Edinburgh, Edinburgh, United Kingdom

**Keywords:** antiviral immunity, cGAS-STING pathway, PRRs, RLRs, TLRs

## Abstract

Viral infectious diseases pose a persistent challenge to global livestock production, animal welfare, and food security, emphasizing the critical role of early host defense mechanisms in limiting viral replication and transmission. As frontline sensors of infection, pattern recognition receptors (PRRs) detect viral nucleic acids and trigger antiviral innate immune responses via coordinated downstream signaling. This review summarizes the functions of three major PRR families, Toll-like receptors (TLRs), RIG-I-like receptors (RLRs), and the cyclic GMP-AMP synthase-stimulator of interferon genes (cGAS-STING) pathway, emphasizing their involvement in detecting viral agents and activating downstream pathways, including NF-κB and IRF3/7. Particular attention is given to how these receptors function in pigs and poultry, highlighting their immune responses to economically significant viruses such as porcine reproductive and respiratory syndrome virus (PRRSV), African swine fever virus (ASFV), avian influenza virus (AIV), and Newcastle disease virus (NDV). The mechanisms by which PRRs activate interferon-mediated immune responses, as well as viral strategies to evade detection, are systematically discussed. Additionally, the review explores recent advances in understanding PRR signaling specificity across species, and their potential applications in vaccine adjuvant design or antiviral drug development are also reviewed. By integrating these insights, this work provides a theoretical foundation for improving disease prevention and control in livestock production.

## 1 Introduction

Viral diseases remain a major challenge to livestock production, particularly in swine and poultry, posing serious risks to animal health, agricultural sustainability, and the global economy (1, 2). Notable emerging and re-emerging pathogens, such as porcine reproductive and respiratory syndrome virus (PRRSV), an enveloped positive-sense RNA virus (3), African swine fever virus (ASFV), a large double-stranded DNA virus (4), avian influenza virus (AIV), an orthomyxovirus with a segmented negative-sense RNA genome (5), and Newcastle disease virus (NDV), a paramyxovirus with a negative-sense RNA genome, are responsible for substantial illness, high mortality rates, and considerable financial losses in these industries (6). Despite extensive vaccination programs and improved biosecurity, high mutation rates of RNA viruses and immune evasion strategies of both RNA and DNA viruses challenge effective long-term control (7).

The innate immune system in vertebrates serves as the first line of defense against viral infections, mounting rapid, non-specific responses and priming adaptive immunity (8, 9). A pivotal element of the host’s antiviral defense is the activity of pattern recognition receptors (PRRs), which sense pathogen-associated molecular patterns (PAMPs), including viral nucleic acids such as RNA and DNA, and initiate downstream antiviral signaling pathways (10, 11). Among the PRR families implicated in antiviral immunity, Toll-like receptors (TLRs), retinoic acid-inducible gene I-like receptors (RLRs), and the cyclic GMP-AMP synthase-stimulator of interferon genes (cGAS-STING) axis are the most extensively characterized (2, 12). TLRs, located on the cell surface or endosomal membranes, recognize viral nucleic acids and activate MyD88- or TRIF-dependent pathways, inducing NF-κB and interferon regulatory factors (IRF3, IRF7) to promote interferon production (13). RLRs such as RIG-I and MDA5 sense cytoplasmic viral RNA and signal via mitochondrial antiviral signaling protein (MAVS) to induce type I interferons and proinflammatory cytokines (14, 15). The cGAS-STING axis detects cytosolic DNA, activating IRF3-mediated antiviral responses through cGAMP production and STING/TBK1 activation (16). Importantly, although these PRR pathways are evolutionarily conserved, their functional contributions differ between pigs and poultry due to species-specific receptor repertoires and signaling capacities. For example, pigs possess a complete RIG-I/MDA5 system for cytosolic RNA sensing, whereas chickens lack functional RIG-I and rely more heavily on MDA5 and endosomal TLRs, while DNA virus recognition via the cGAS-STING pathway is particularly prominent in pigs during ASFV infection (17).

In pigs, PRRSV, a positive-sense RNA virus, is mainly sensed by RIG-I and MDA5; however, it encodes several non-structural proteins (nsp1α, nsp1β, nsp2, nsp5) that inhibit MAVS, TBK1, and IRF3 signaling, suppressing IFN-β production and enabling immune evasion (17). ASFV, a large double-stranded DNA virus, is primarily detected by the cGAS-STING pathway, yet encodes proteins like MGF360-11L and MGF505-7R that inhibit DNA sensing and the STING-TBK1-IRF3 axis, blocking type I interferon production (18, 19). Poultry innate immune recognition differs due to genetic variations; chickens lack functional RIG-I and rely more on MDA5 and TLR3/TLR7 for AIV and NDV detection. AIV RNA is mainly recognized by TLR7 and MDA5, although highly pathogenic strains express NS1 protein that suppresses MAVS and IRF3 activation (20). NDV is sensed by MDA5 and TLR3 but its V protein antagonizes IFN signaling by targeting STAT1 or IRF7, facilitating viral persistence (21–23).

**Figure 1:**
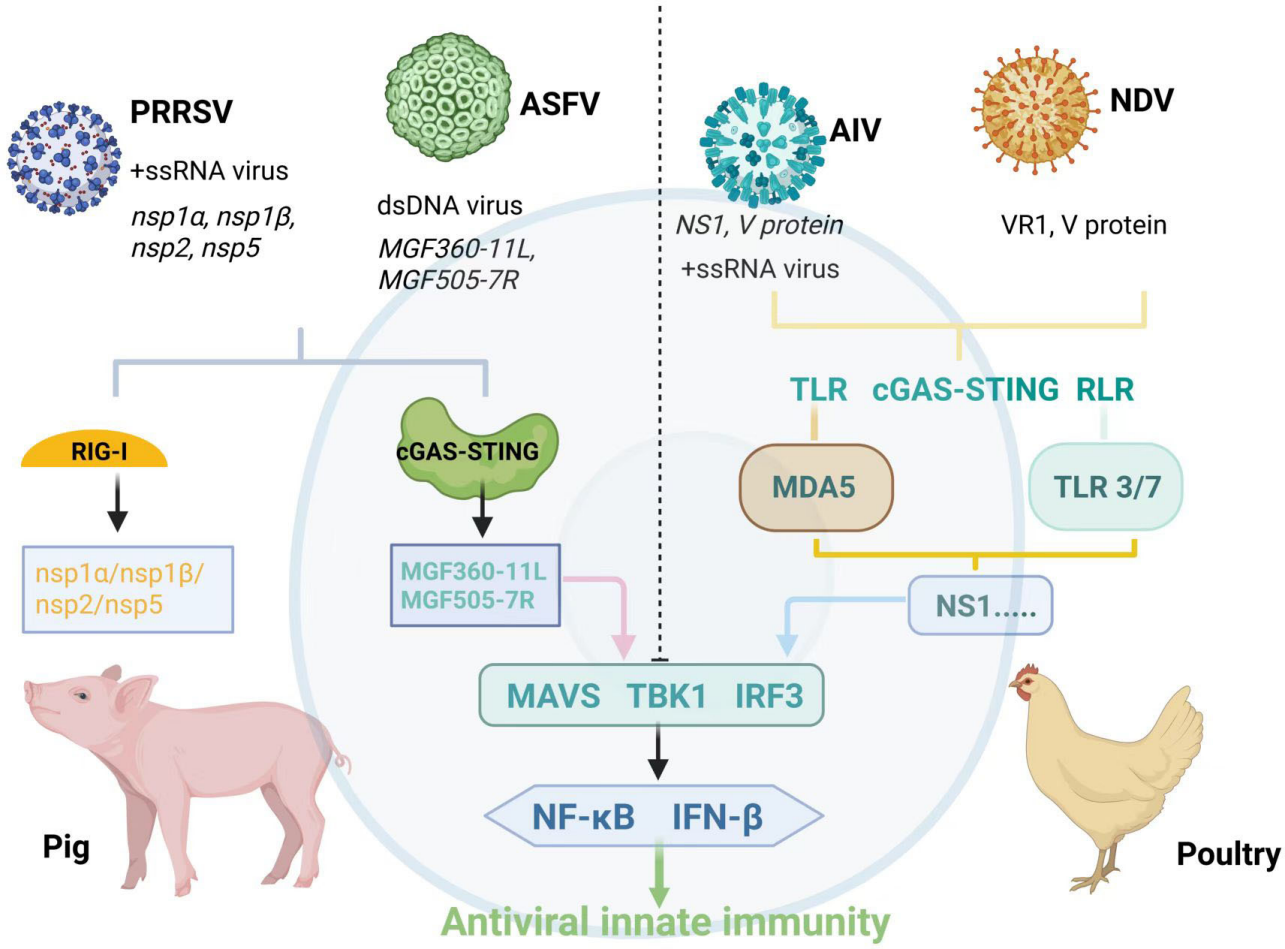
Recognition of viral pathogens by PRRs and immune evasion mechanisms in pigs and poultry. This figure summarizes how major livestock viruses are recognized by distinct PRRs and how they subvert host innate immunity. TLRs, RLRs, and the cGAS-STING pathway detect viral nucleic acids in different cellular compartments. Endosomal TLR3 and TLR7 respond to viral RNA, cytosolic RIG-I and MDA5 recognize RNA species generated during viral replication, while the cGAS-STING axis detects viral DNA. Representative viruses, including PRRSV, AIV, NDV, and ASFV, activate these pathways but also encode viral proteins (e.g., PRRSV nsp1α/β, nsp2, nsp5; AIV NS1; NDV V; ASFV MGF360 and MGF505) that interfere with key signaling molecules such as MAVS, TBK1, and IRF3. These immune evasion strategies ultimately impair IFN-β production and help viruses escape host antiviral responses. Created with BioRender.com. TLR3, TLR7, and TLR9 are primarily localized to endosomal compartments, whereas RIG-I and MDA5 function as cytosolic RNA sensors. The cGAS-STING pathway is initiated in the cytosol and endoplasmic reticulum, respectively. Representative viruses, including porcine reproductive and PRRSV, AIV, NDV, and ASFV, activate these innate immune pathways but also encode specialized immune evasion proteins. For example, PRRSV nsp1α and nsp1β suppress type I interferon production through distinct mechanisms, with nsp1α promoting CBP degradation and nsp1β inhibiting IRF3 activation and nuclear translocation; nsp2 and nsp5 further antagonize interferon signaling. AIV NS1 blocks viral RNA sensing and host gene expression, NDV V protein targets MDA5-dependent signaling, and ASFV MGF360 and MGF505 family members inhibit interferon induction by disrupting key components of the cGAS-STING and IRF3 pathways.

Despite advances in understanding individual PRRs and their downstream signaling in pigs and poultry, comprehensive cross-species and virus-specific comparisons remain limited (24, 25). The interplay between viral immune evasion and species-specific PRR signaling, as well as the redundancy and cross-talk among PRRs, complicates the antiviral landscape (26). For example, RIG-I and MDA5 may compensate for each other in certain contexts but differ in expression and activation across species and tissues (25). Moreover, cGAS-STING, traditionally viewed as a DNA sensor, has emerging roles in RNA virus responses and inflammation regulation. Beyond pathogen sensing, PRRs shape broader immune responses and are promising targets for vaccine adjuvants and therapeutics (12). Given the growing impact of viral diseases in livestock and interest in host-directed strategies, this review aims to systematically summarize the roles of TLRs, RLRs, and cGAS-STING in antiviral innate immunity in pigs and poultry, focusing on key viral pathogens (PRRSV, ASFV, AIV, NDV), their evasion tactics, and species-specific signaling. The review is organized into four parts: major viral threats; mechanistic insights into PRR-mediated recognition; virus-specific interactions and immune evasion; and cross-species comparisons and translational perspectives. This synthesis aims to facilitate the development of innovative antiviral approaches harnessing innate immunity in livestock. The structure of this review is organized as follows (**Figure 1**): (1) an overview of major viral threats in pigs and poultry; (2) mechanistic insights into TLR, RLR, and cGAS-STING mediated recognition; (3) virus-specific interactions and immune evasion strategies; (4) cross-species comparisons and translational implications. By synthesizing the latest findings, we aim to support the development of next-generation antiviral interventions that harness the power of innate immunity in livestock. This review thoroughly summarizes the latest advances in pattern recognition receptors and their downstream signaling pathways in key livestock species, while also identifying current knowledge gaps and future research directions. By exploring the complex interactions between viral pathogens and the host innate immune system, especially in economically important animals such as pigs and poultry, this work aims to provide a solid foundation for disease control strategies. Ultimately, it seeks to guide the development of novel antiviral therapies and vaccines that harness innate immunity, contributing to improved animal health and sustainable livestock production.

## 2 Classification and fundamental mechanisms of PRRs

In livestock antiviral innate immunity, PRRs serve as the primary sentinels detecting invading viral pathogens. These receptors are broadly categorized into three major families, TLRs, RLRs, and the cGAS-STING pathway, each distinguished by their ligand specificities, subcellular localization, and downstream signaling mechanisms. To provide a clear overview of their classification and fundamental roles in pigs and poultry, **Table 1** summarizes the key receptors, their localization, recognized viral components, adaptor proteins involved, and notable species-specific differences.

**Table 1:** Summary of PRRs in pigs and poultry.

PRR type	Key receptors	Recognized ligands	Signaling adaptors	Key features in pigs	Key features in poultry
Toll-like Receptors (TLRs)	TLR1/2/4/5/6 (surface); TLR3/7/8/9 (endosome)	TLR3: dsRNATLR7/8: GU-rich ssRNATLR9: CpG DNA	MyD88 (TLR7/8/9)TRIF (TLR3)	TLR7 & TLR9 show tissue-specific expression; respond to PRRSV, AIV, ASFV	TLR7 rapidly upregulated during ssRNA virus infection; poly(I:C) and imidazoquinoline used as adjuvants
RIG-I-like Receptors (RLRs)	RIG-I, MDA5, LGP2	RIG-I: 5’-triphosphate RNA, short blunt dsRNAMDA5: long dsRNA	MAVS	Possess both RIG-I and MDA5; enable broad RNA virus recognition (e.g., PRRSV, CSFV)	Lacks functional RIG-I; MDA5 compensates, senses both short & long dsRNA (e.g., AIV)
cGAS–STING Pathway	cGAS, STING	Cytosolic double-stranded DNA (dsDNA)	STING → TBK1 → IRF3/7	Upregulated during ASFV infection; ASFV proteins (MGF505-7R, I215L) inhibit TBK1/STING	cGAS/STING orthologs identified; overexpression enhances IFN-β; knockdown reduces antiviral genes

Notably, the repertoire of RLR genes differs markedly among livestock species. Pigs possess a complete RLR system comprising RIG-I, MDA5, and LGP2, enabling broad and layered recognition of viral RNA species. In contrast, chickens lack a functional RIG-I gene and therefore rely predominantly on MDA5-mediated cytosolic RNA sensing. Ducks, however, retain both RIG-I and MDA5, allowing more rapid and robust activation of antiviral signaling pathways upon influenza virus infection (17, 27). These gene presence/absence differences constitute a fundamental molecular basis for species-specific antiviral susceptibility and immune competence.

### 2.1 Toll-like receptors

TLRs are evolutionarily conserved pattern recognition receptors that play a pivotal role in detecting viral pathogens and initiating innate immune responses (2). TLRs can be categorized according to their location within the cell, with one major group positioned on the plasma membrane, including TLR1, TLR2, TLR4, TLR5, and TLR6, which primarily recognize bacterial and viral envelope components, and those localized within endosomal membranes (e.g., TLR3, TLR7/8, TLR9), which sense nucleic acids derived from internalized viruses or dying cells (28).

Each endosomal TLR specializes in detecting distinct viral nucleic acid patterns. TLR3 recognizes viral double-stranded RNA (dsRNA), a replicative intermediate generated by many RNA viruses, including NDV and AIV (29). This MyD88-independent, TRIF-dependent signaling endows TLR3 with a unique functional role in antiviral immunity by enabling robust type I interferon production while limiting excessive proinflammatory cytokine responses. Such signaling bias is particularly advantageous during viral infection, as it promotes effective antiviral states without exacerbating immunopathology. TLR7 and TLR8 sense GU-rich single-stranded RNA (ssRNA) from viruses such as PRRSV and IAV, while TLR9 detects unmethylated CpG motifs commonly found in the DNA genomes of viruses like ASFV (30).

Downstream signaling pathways of TLRs are mediated by adaptor proteins, primarily MyD88 and TRIF, which determine the nature and kinetics of immune responses (31). Most TLRs, such as TLR7, TLR8, and TLR9, primarily engage the MyD88-dependent signaling route. This activation subsequently triggers NF-κB and IRF7, driving the production of pro-inflammatory cytokines and type I interferons (32). In contrast, TLR3 uniquely signals via the TRIF-dependent pathway, triggering IRF3-mediated interferon production without MyD88 involvement, a critical feature for antiviral responses to dsRNA viruses (33).

Recent comparative studies in pigs and chickens have highlighted both conserved and divergent roles of TLRs (17, 34, 35). For example, chicken TLR7 exhibits rapid upregulation upon ssRNA virus infection and contributes significantly to the early antiviral cytokine burst, whereas porcine TLR7 and TLR9 display tissue-specific expression profiles and temporal differences in response to AIV or ASFV, indicating species-adapted immune regulation (36).

Furthermore, vaccine strategies leveraging TLR signaling have gained attention. Use of TLR3 and TLR7 agonists in poultry and swine vaccines enhances both humoral and cellular immunity. In chickens, co-delivery of poly(I:C) (TLR3 agonist) and imidazoquinolines (TLR7 agonist) with inactivated NDV or AIV antigens has shown synergistic enhancement of immune responses, suggesting combinatorial adjuvant strategies can improve vaccine efficacy (37–39).

In summary, TLRs serve as key antiviral sentinels, with their localization, ligand specificity, and adaptor-mediated signaling underpinning their functional diversity. Cross-species comparison reveals both conserved core pathways and species-specific adaptations, providing insights for the rational design of PRR-targeted therapeutics and adjuvants in livestock immunology.

### 2.2 RIG-I-like receptors

RIG-I-like receptors (RLRs), comprising RIG-I, MDA5, and LGP2, represent critical cytoplasmic sensors for viral RNA, orchestrating antiviral innate immunity in livestock species such as pigs and poultry (40). While both pigs and chickens rely on RLRs to detect RNA virus infections, there are notable species-specific differences in receptor repertoire and functionality that influence their antiviral responses (27, 41, 42).

In pigs, RIG-I and MDA5 distinctly recognize viral RNA species (15, 25, 42, 43): RIG-I preferentially binds short 5’-triphosphate RNA and blunt-ended dsRNA, commonly generated during negative-strand RNA virus replication, whereas MDA5 senses long dsRNA intermediates typical of positive-strand RNA viruses. This complementary recognition expands the breadth of RNA virus detection in pigs, which is pivotal for mounting timely interferon responses against pathogens like porcine reproductive and PRRSV and CSFV (44). In contrast, chickens notably lack a functional RIG-I ortholog, relying predominantly on MDA5 for cytosolic RNA sensing (45, 46). This evolutionary loss compels poultry to compensate through MDA5-mediated recognition of both short and long viral RNA motifs, which partially explains differential susceptibility patterns to RNA viruses such as AIV compared to pigs.

Taken together, the comparative analysis of RLR-mediated RNA virus recognition and MAVS signaling in pigs and poultry highlights evolutionary divergence shaping innate immune strategies. While pigs exhibit a classical dual RIG-I/MDA5 system facilitating nuanced viral RNA sensing, chickens rely heavily on MDA5 and possibly other PRRs to orchestrate antiviral defenses. Understanding these differences provides insights for tailored antiviral and vaccine strategies in these key livestock species.

### 2.3 The cGAS-STING pathway

The cGAS)- STING pathway has emerged as a pivotal mechanism for the cytosolic detection of DNA viruses in vertebrates, including key livestock species such as pigs and poultry (47). When double-stranded DNA (dsDNA) is detected in the cytoplasm, cyclic GMP-AMP synthase (cGAS) produces the second messenger cyclic GMP-AMP (cGAMP). This molecule then interacts with and activates the adaptor protein STING, located on the endoplasmic reticulum membrane (48). Once activated, STING recruits TANK-binding kinase 1 (TBK1), which in turn phosphorylates interferon regulatory factors IRF3 and IRF7. The activated IRFs drive the transcription of type I interferons and a range of antiviral genes (49).

In pigs, the cGAS-STING axis plays a crucial role in the innate immune response to ASFV, a large cytoplasmic DNA virus with sophisticated immune evasion strategies (50). Recent studies have demonstrated that cGAS and STING are both transcriptionally upregulated following ASFV infection in porcine alveolar macrophages, and genetic ablation or inhibition of either molecule results in impaired type I IFN responses and enhanced viral replication (51). However, ASFV encodes multiple proteins (e.g., MGF505-7R, I215L) that actively disrupt the cGAS-STING signaling cascade by targeting TBK1 and STING, allowing the virus to blunt host interferon responses (52).

In contrast, research on cGAS-STING signaling in poultry remains comparatively limited, yet emerging evidence highlights its relevance in defense against avian DNA pathogens such as Duck Tembusu virus and Riemerella anatipestifer, a bacterium with intracellular DNA sensing implications (53). Notably, functional cGAS and STING orthologs have been identified in ducks and chickens, and their overexpression *in vitro* enhances IFN-β production upon DNA virus challenge, while knockdown experiments diminish antiviral gene expression (54, 55). The evolutionary conservation of this pathway, despite sequence divergence, suggests that poultry utilize cGAS-STING as a frontline defense mechanism against DNA viruses, although further *in vivo* validation is warranted.

## 3 Downstream signaling pathways of PRRs and interferon responses

### 3.1 The NF-κB signaling pathway

The NF-κB signaling pathway plays a pivotal role downstream of PRRs in coordinating antiviral innate immune responses in livestock such as pigs and poultry (56, 57). When viral ligands are detected, adaptor proteins such as MyD88 and TRIF initiate downstream signaling events that activate the IκB kinase (IKK) complex. This complex is primarily composed of the catalytic subunits IKKα and IKKβ, along with the regulatory component NEMO (also known as IKKγ) (43, 58, 59). The activated IKK complex phosphorylates the inhibitor proteins IκBs, targeting them for ubiquitination and subsequent degradation. This process releases NF-κB dimers, primarily p65/p50, that then translocate into the nucleus to initiate transcription (60, 61). Upon activation, NF-κB drives the transcription of pivotal proinflammatory mediators, including TNF-α and IL-6. These cytokines play a central role in orchestrating a robust inflammatory response necessary for controlling viral infections (62). Research using porcine alveolar macrophages has shown that NF-κB activation not only triggers the production of inflammatory cytokines but also promotes type I interferon responses, thereby establishing an antiviral state (63). Similar mechanisms have been observed in chickens, although differences in the timing and level of cytokine production may underlie species-specific variations in antiviral defense (64). Moreover, negative regulators such as A20 and CYLD modulate NF-κB signaling to prevent excessive inflammation and tissue damage during viral challenges in both species (65). Deciphering these regulatory pathways is vital for developing strategies that enhance antiviral immunity while minimizing immunopathology in livestock.

### 3.2 IRF3/IRF7 signaling pathway

Activation of PRRs triggers the phosphorylation and nuclear translocation of interferon regulatory factors 3 and 7 (IRF3/7), which are pivotal transcription factors driving type I interferon (IFN-α/β) production, a cornerstone of antiviral innate immunity in livestock such as pigs and poultry (66, 67). Upon viral RNA or DNA recognition, adaptor proteins such as MAVS, STING, MyD88, or TRIF facilitate the recruitment and activation of kinases TBK1 and IKKϵ, which phosphorylate IRF3/7, enabling their dimerization and nuclear import to initiate IFN gene transcription (68). In pigs, robust activation of IRF3 and IRF7 has been documented during infections with RNA viruses like porcine reproductive and PRRSV and CSFV, correlating with potent type I IFN responses critical for viral control (69). Conversely, chickens predominantly rely on IRF7 due to the absence of RIG-I, and studies suggest differential kinetics and intensity of IRF7 activation compared to pigs, which may underlie species-specific variations in antiviral defenses against pathogens such as AIV (70). The type I interferons subsequently induce a wide array of ISGs, including Mx, OAS, and PKR, which mediate diverse antiviral effects ranging from viral replication inhibition to apoptosis of infected cells (71). Notably, comparative transcriptomic analyses have revealed both conserved and divergent patterns of ISG induction between pigs and poultry, reflecting evolutionary adaptations to their respective viral threats and immune system architectures (72, 73). Together, these findings underscore the centrality of IRF3/7-dependent signaling in orchestrating type I IFN responses in livestock, while highlighting species-specific nuances that could inform tailored antiviral strategies and vaccine development.

### 3.3 JAK-STAT pathway and ISG expression

Upon secretion, type I interferons (IFN-α/β) bind to their specific surface receptors, initiating the Janus kinase (JAK), signal transducer and activator of transcription (STAT) signaling pathway (74, 75). In both swine and poultry, receptor engagement activates the associated kinases JAK1 and TYK2, which in turn phosphorylate STAT1 and STAT2 (76, 77). The phosphorylated STAT proteins heterodimerize and recruit interferon regulatory factor 9 (IRF9), forming the interferon-stimulated gene factor 3 (ISGF3) complex. Once assembled, ISGF3 translocates into the nucleus and binds to interferon-stimulated response elements (ISREs) in target gene promoters, driving the transcription of interferon-stimulated genes (ISGs) that mediate antiviral defense (78, 79).

The ISGs induced through JAK-STAT signaling constitute a broad antiviral arsenal, including canonical effectors such as Mx1, OAS, and ISG15, which execute diverse functions from inhibition of viral replication and assembly to modulation of host cell apoptosis and immune regulation (80, 81). In pigs, the robust induction of these ISGs has been closely associated with effective control of viruses like porcine reproductive and PRRSV and CSFV, where defects or viral antagonism of JAK-STAT signaling correspond with increased viral persistence and pathogenicity (78, 82, 83).

Comparatively, poultry also rely on this pathway for mounting antiviral defenses, although subtle differences in STAT protein isoforms and regulatory mechanisms may influence the magnitude and kinetics of ISG expression (84, 85). For example, variations in the expression levels of STAT2 and IRF9 in chickens relative to pigs have been linked to differential susceptibility to viruses such as AIV, suggesting evolutionary adaptation of the JAK-STAT axis to distinct viral landscapes (84, 86). Moreover, viral pathogens in poultry have evolved specific antagonistic strategies targeting JAK-STAT components, underscoring the critical role of this pathway in antiviral immunity.

To conclude, the JAK-STAT pathway plays a central role in converting interferon signals into a well-orchestrated antiviral gene response in livestock. In particular, key distinct features include species-specific PRR repertoires (such as the presence or absence of RIG-I), differential STAT isoform usage and signaling efficiency, distinct kinetic profiles of interferon-stimulated gene expression, and virus-specific immune evasion strategies targeting PRR signaling pathways.

## 4 Recognition mechanisms and immune evasion of viruses in pigs and poultry

To better illustrate the interplay between host recognition mechanisms and viral immune evasion strategies, especially in economically important livestock species, we summarize below the key PRRs, viral sensing pathways, and immune evasion tactics employed by representative RNA and DNA viruses in pigs and poultry. This comparative overview highlights species-specific differences in innate immune recognition and provides mechanistic insights into why certain viruses establish more persistent or severe infections in specific hosts (**Table 2**). Collectively, PRRSV primarily evades host immunity by suppressing interferon induction at multiple signaling nodes, ASFV by targeting the cGAS-STING axis, AIV by NS1-mediated inhibition of RNA sensing and host gene expression, and NDV by V protein–mediated antagonism of MDA5 and downstream interferon signaling.

**Table 2:** Viral recognition mechanisms and immune evasion strategies in pigs and poultry.

Aspect	Species	Virus type	Key PRRs	Recognition pathways	Viral immune evasion strategies
PRRSV recognition	Pig	RNA virus (PRRSV)	TLR7, RIG-I	TLR7 senses ssRNA in endosomes; RIG-I detects cytosolic RNA → Activates IRF3 → Type I IFN production	PRRSV nsp1 and nsp11 inhibit IRF3 activation and degrade signaling intermediates to suppress IFN responses
ASFV recognition	Pig	DNA virus (ASFV)	cGAS-STING	cGAS detects cytosolic dsDNA → Activates STING → TBK1 → IRF3 → Type I IFN production	ASFV proteins (MGF360/505) inhibit STING activation or downstream signaling to block host defenses
IAV recognition	Duck	RNA virus (Influenza A)	MDA5, RIG-I	MDA5 detects viral dsRNA → MAVS → TBK1/IRF7 → Strong type I IFN response	Ducks show natural resistance to IAV; major immune evasion not described
NDV/IAV recognition	Chicken	RNA virus (NDV, IAV)	TLR3, TLR7, MDA5 (no RIG-I)	TLR3 detects dsRNA, TLR7 senses GU-rich ssRNA; MDA5 partially compensates → Type I IFN response	Absence of RIG-I leads to delayed and weaker IFN responses, increasing viral susceptibility

### 4.1 Viral recognition mechanisms in pigs

While the core PRR signaling architecture is broadly conserved across vertebrates, species-specific variations in receptor repertoire, signaling strength, and regulatory mechanisms critically shape antiviral immunity in livestock. In pigs, the recognition of viral pathogens predominantly involves several key PRRs that detect distinct viral components to initiate innate immune responses (15). Porcine reproductive and PRRSV, a major RNA virus afflicting swine populations globally, is primarily sensed by endosomal TLR7 and cytoplasmic RIG-I-like receptors, notably RIG-I (87, 88). Stimulation of these receptors generally triggers the production of type I interferons along with proinflammatory cytokines, both of which are crucial for suppressing viral proliferation (89). However, PRRSV has evolved sophisticated immune evasion strategies that suppress interferon signaling pathways. For example, PRRSV nonstructural proteins such as nsp1 and nsp11 actively interfere with IRF3 activation and degrade key signaling molecules, effectively dampening the antiviral IFN response and facilitating viral persistence (90, 91).

**Figure 2:**
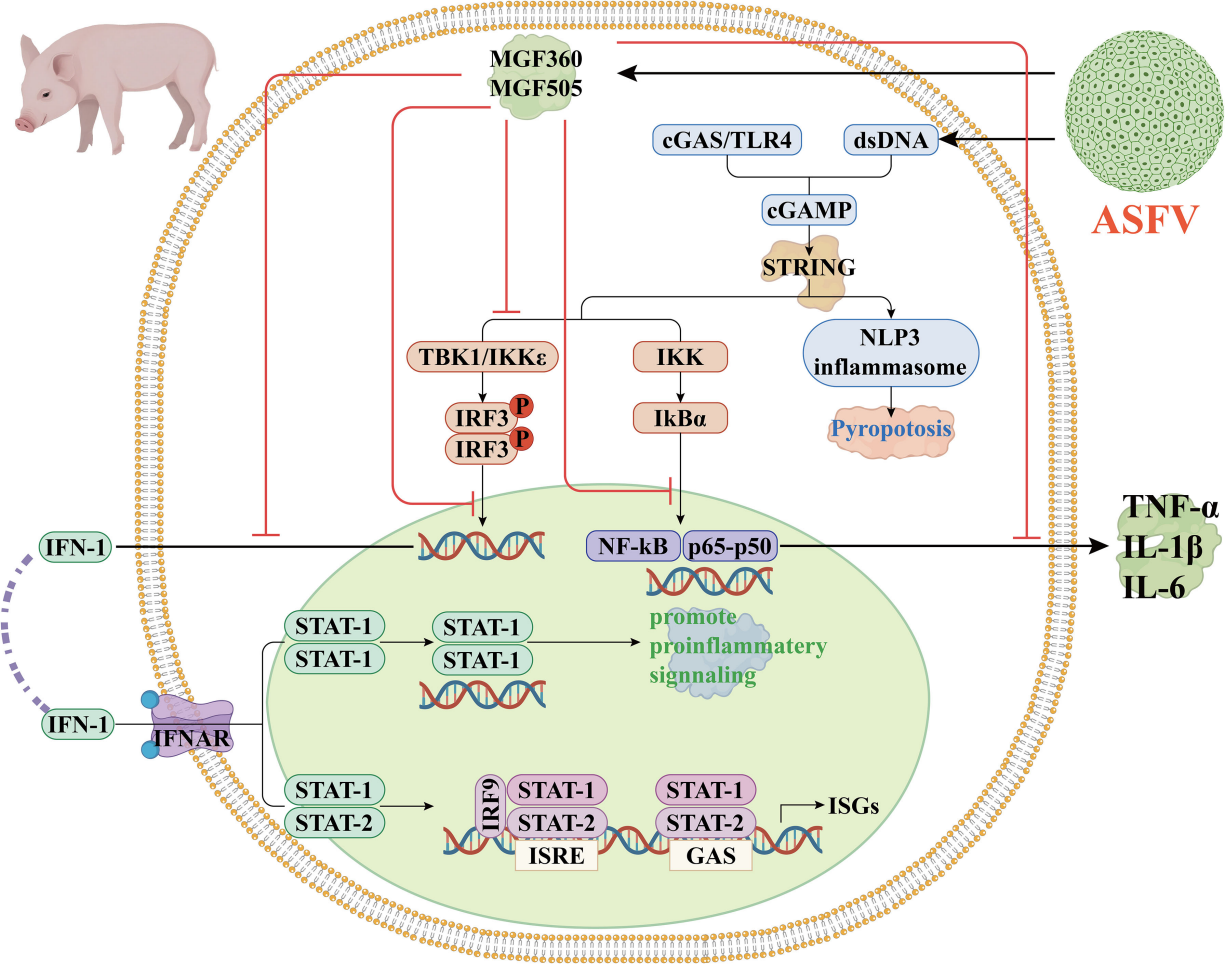
Host innate immunity during ASFV infection. ASFV primarily targets porcine macrophages, where it is detected by both membrane-bound and PRRs. Recognition of viral components triggers diverse signaling pathways that converge on transcription factors like NF-κB and IRF3, which in turn promote the production of interferons and inflammatory mediators. Binding of IFNs to their receptors activates downstream signaling, notably through the canonical STAT1-STAT2 heterodimer, which associates with interferon-stimulated response elements to induce a broad spectrum of ISGs. In parallel, STAT1 homodimers promote proinflammatory signaling. ASFV infection also triggers activation of the NLRP3 inflammasome, leading to pyroptotic cell death. However, ASFV has evolved multiple strategies to suppress host immunity: several viral proteins (indicated in red) disrupt IFN and cytokine production during early transcriptional events. Importantly, the degree to which ASFV impairs antigen presentation in macrophages appears to vary among viral strains.

ASFV encodes multiple immune evasion proteins that directly target key components of host innate immune signaling. For instance, the viral protein pE248R interferes with cGAS-STING signaling by disrupting STING activation, whereas MGF360 and MGF505 family members suppress type I interferon production by inhibiting IRF3 phosphorylation and nuclear translocation (**Figure 2**) (50, 92, 93).

These dual examples highlight how porcine viruses have adapted to both utilize and evade PRR-mediated immune recognition, underscoring the dynamic interplay between host sensing mechanisms and viral countermeasures.

### 4.2 Viral recognition mechanisms in poultry

In poultry, the detection of viral pathogens is mediated by a repertoire of PRRs that exhibit both conserved and divergent features compared to their mammalian counterparts. Notably, ducks and chickens, two key representatives of poultry livestock, show distinct immune receptor expression profiles that significantly influence their antiviral innate immune responses (94, 95).

Ducks are known for their exceptional resilience to IAV, a trait largely attributed to the efficient recognition of viral dsRNA intermediates by MDA5 (27, 95). Unlike mammals, ducks lack the RIG-I gene, and MDA5 compensates by acting as the dominant cytosolic sensor of IAV, leading to downstream activation of MAVS, TBK1, and IRF7, and robust induction of type I interferons (95). This mechanism contributes to the early control of viral replication and explains, in part, the relatively asymptomatic IAV infection course observed in ducks compared to chickens (96). Beyond the presence of RIG-I, ducks display distinct immune regulatory features that contribute to their natural resistance to AIV. Duck RIG-I and MDA5 signaling is characterized by rapid induction of type I interferons with relatively restrained proinflammatory cytokine production, thereby limiting immunopathology. In addition, duck-specific differences in negative regulatory pathways, interferon feedback control, and antiviral ISG expression patterns have been proposed to promote effective viral clearance while avoiding excessive tissue damage (73, 84). Additional contributing factors may include differential expression of antiviral ISGs, enhanced basal interferon readiness, and evolutionary adaptation to aquatic virus reservoirs, all of which likely cooperate with PRR-mediated sensing to confer heightened AIV resistance in ducks.

In contrast, chickens exhibit a reduced PRR repertoire, most notably the absence of functional RIG-I and TLR8 orthologs (84). This structural limitation is associated with a heightened susceptibility to certain RNA viruses and necessitates compensatory reliance on alternative sensors such as TLR3 and TLR7, which are localized to endosomes and recognize viral dsRNA and GU-rich single-stranded RNA, respectively (84). For instance, infection with NDV triggers robust expression of TLR3 and TLR7 in chicken splenocytes and macrophages, leading to the production of type I IFNs and inflammatory cytokines (97). Despite lacking RIG-I, chickens utilize MDA5 to some extent in sensing NDV and IAV, though the magnitude and timing of the response differ markedly from that in ducks.

Interestingly, the functional absence of RIG-I in chickens correlates with delayed and reduced interferon responses following RNA virus infection, a factor that may partially explain their greater clinical vulnerability. Conversely, ducks retain both RIG-I and MDA5, allowing for a more layered and robust antiviral signaling cascade. The evolutionary divergence of PRRs in poultry species thus underscores an important axis of differential viral susceptibility and offers insights into species-specific immune tuning (27).

Altogether, poultry innate immune recognition mechanisms reflect a complex interplay between conserved PRRs and species-specific adaptations. A clearer understanding of these differences not only informs comparative immunology but also supports the rational design of immunomodulatory interventions and genetic selection strategies in poultry farming.

### 4.3 Viral immune evasion strategies

To achieve successful infection and replication, many viruses employ diverse strategies to evade the host’s innate immune defenses. These tactics frequently target pattern recognition receptor (PRR) signaling, a central element of early antiviral immunity responsible for initiating interferon production. In both pigs and poultry, various viruses exploit vulnerabilities in these signaling networks to impair immune detection and response (44, 98).

A primary mode of evasion is the direct targeting of PRRs, either by suppressing their expression or promoting their degradation, thereby hindering the host’s ability to sense viral nucleic acids. For example, the NS1 protein of IAV in ducks and chickens can antagonize the activity of RIG-I or MDA5 by blocking their activation or interfering with their interactions within the signaling complex (95). Similarly, PRV and porcine reproductive and PRRSV encode proteins such as UL13 and nsp1α that promote degradation of RIG-I or cGAS in pigs, thereby attenuating the initial antiviral signaling triggered by viral RNA or DNA (89, 99).

Beyond receptor-level interference, viruses often disrupt key downstream signaling events, particularly those involving the activation of IRF transcription factors (44). In pigs, PRRSV nsp1β has been shown to inhibit TBK1-mediated phosphorylation of IRF3, a critical step for IFN gene induction. Porcine circovirus type 2 (PCV2) similarly impairs antiviral signaling by targeting both TBK1 and IKKϵ (100, 101). In poultry, the V protein of NDV interferes with the MDA5-MAVS axis by sequestering MDA5 or LGP2, preventing MAVS activation and downstream signaling events that lead to IFN production (21).

Viruses also manipulate host protein synthesis and degradation as an additional layer of immune evasion. In swine, PRRSV nsp2 and nsp11 have been reported to degrade STAT2 or inhibit host mRNA translation, thereby dampening the expression of ISGs (102). Influenza A virus employs a similar tactic: its NS1 protein inhibits host mRNA processing, effectively shutting down host protein synthesis and reducing the availability of antiviral effectors (103, 104). In avian species, certain viruses exploit the ubiquitin, proteasome system to degrade signaling intermediates such as MAVS and TRAF3, further impairing immune activation.

These multifaceted strategies reflect a dynamic evolutionary arms race between viruses and their hosts. Despite differences in PRR repertoires between pigs and poultry, many viruses have evolved both conserved and host-specific mechanisms to subvert innate immune detection. Elucidating these evasion tactics not only enhances our understanding of viral pathogenesis but also provides critical insights for the development of more effective vaccines and antiviral therapies in livestock.

## 5 Practical applications and future perspectives

Given their central role in triggering innate immune defenses, PRRs have emerged as attractive targets for the development of antiviral therapeutics and vaccine adjuvants in livestock species. Recent studies have demonstrated that stimulation of PRRs can significantly amplify both humoral and cellular immune responses, thereby enhancing vaccine efficacy beyond that achieved by conventional formulations. In swine and poultry, synthetic PRR agonists have been explored as adjunct components in vaccination programs (105). For instance, polyinosinic:polycytidylic acid [poly(I:C)], a double-stranded RNA mimic acting as a TLR3 agonist, has been incorporated into experimental swine vaccines, leading to enhanced antigen presentation, increased interferon production, and prolonged antibody titers against PRRSV. Similarly, imidazoquinoline derivatives, which activate TLR7, have been evaluated in poultry immunization against NDV, resulting in stronger mucosal immunity and more rapid viral clearance upon challenge (106). Beyond these examples, a growing body of evidence indicates that carefully selected PRR agonists not only improve the magnitude of immune responses but also fine-tune their quality, promoting balanced Th1/Th2 polarization and durable memory responses. This immunomodulatory capacity is particularly valuable in livestock production, where vaccines must induce long-lasting protection under field conditions characterized by high pathogen pressure and varied environmental stressors. Beyond vaccine adjuvants, PRR agonists hold potential as therapeutic or management tools, including early antiviral intervention, enhancement of mucosal immunity, and reduction of viral shedding during outbreaks. Such strategies may complement vaccination programs and contribute to integrated disease control in livestock production systems. Despite their promise, several challenges limit the widespread application of PRR agonists in livestock. These include species-specific differences in PRR responsiveness, the risk of excessive inflammation, limited understanding of optimal dosing and delivery routes, and potential interference with existing immune homeostasis. Addressing these gaps will be essential for the rational and safe deployment of PRR-based immunomodulatory strategies.

Beyond these pharmacological interventions, advancements in genome editing have opened new possibilities for bolstering innate antiviral resistance by directly modulating PRR pathways. Notably, transgenic chickens engineered to express RIG-I, which is naturally missing in Galliformes, exhibit heightened detection of RNA viruses such as AIV, leading to elevated type I interferon responses and reduced viral replication upon infection (17, 27). This approach illustrates how genetic enhancement of PRRs can restore critical antiviral functions lost through evolution, thereby altering species-specific susceptibility patterns.

The application of high-throughput omics technologies, including transcriptomics, proteomics, and single-cell analyses, has started to shed light on novel regulatory mechanisms governing PRR signaling and downstream interferon-stimulated gene networks in livestock (69, 107). Recent integrative studies in pigs and chickens have uncovered previously unrecognized adaptor proteins, microRNAs, and epigenetic modifications that influence PRR activity during viral challenges, emphasizing the complexity and species-specific nuances of innate immune regulation. These findings provide valuable avenues for designing precise immunomodulatory strategies that enhance antiviral defense while limiting excessive inflammation.

Despite growing insights into PRR-mediated antiviral immunity, important knowledge gaps remain regarding the functional diversity of these receptors across livestock species. Comparative studies have shown that while the core signaling pathways are evolutionarily conserved, pigs and poultry display notable differences in PRR gene repertoires, activation kinetics, and tissue-specific expression patterns. These distinctions have a profound impact on how each species detects and responds to viral infections. A deeper understanding of such interspecies variation, enabled by integrated immunogenomic approaches, will be key to developing more effective, species-adapted vaccines and immunomodulatory strategies.

Looking ahead, PRRs represent a promising avenue for improving antiviral defenses in livestock, particularly when leveraged alongside advances in genome editing and systems-level analyses. Future research should aim to clarify the molecular basis of PRR variation, refine adjuvant strategies to align with host-specific immune contexts, and expand the application of gene-editing tools to enhance disease resistance. These multidisciplinary efforts will be critical for strengthening livestock resilience, reducing the burden of viral disease, and ensuring the sustainability of animal agriculture in the face of evolving pathogen threats.

## 6 Conclusion

PRRs are essential components of the innate immune system, acting as the first line of defense against viral pathogens by detecting viral nucleic acids and triggering type I interferon production and proinflammatory responses. In pigs and poultry, PRRs not only initiate early antiviral mechanisms but also influence the development and magnitude of adaptive immune responses. This dual role makes them attractive targets for vaccine development and antiviral strategies in livestock.

Notable progress has been made in recent years toward mapping the PRR network in pigs. For example, endosomal TLR7 and cytosolic RIG-I-like receptors have been shown to recognize RNA viruses such as porcine reproductive and respiratory syndrome virus (PRRSV), while the cGAS-STING pathway plays a crucial role in sensing DNA viruses like ASFV. In contrast, avian species present a different immunological profile, partly due to gene losses during evolution. Chickens lack key PRRs such as RIG-I and TLR8, which limits their capacity for robust innate sensing of RNA viruses like AIV and NDV. Ducks, however, retain RIG-I and exhibit more rapid and potent interferon responses to AIV, offering a plausible explanation for their relatively higher resistance. These interspecies differences in PRR composition and signaling emphasize the need for comparative approaches tailored to each host’s unique immune architecture.

Despite these advances, considerable knowledge gaps remain. Current studies often rely on *in vitro* models or are restricted to a narrow range of PRRs and immune cell types. The tissue-specific expression and temporal regulation of PRRs during viral infection, particularly in poultry, are still poorly understood. Furthermore, key modulators of PRR signaling, including adaptor proteins, microRNAs, and epigenetic factors, have not been comprehensively characterized in livestock. The application of advanced tools such as single-cell RNA sequencing and integrated omics platforms in farm animals remains limited, hindering a systems-level understanding of antiviral innate immunity. Moreover, cross-species comparative studies are still lacking, which impedes the translation of mechanistic insights into effective interventions.

As viral threats continue to challenge the livestock industry, there is an urgent need to expand our understanding of PRR biology in species-specific contexts. Future research should adopt integrative strategies that combine immunogenomics, systems biology, and precision gene editing. Notably, efforts to restore antiviral function in chickens by reintroducing RIG-I via CRISPR/Cas9-mediated approaches have yielded promising outcomes. Likewise, the use of PRR agonists such as poly(I:C) and imidazoquinolines as vaccine adjuvants has demonstrated potential to enhance both humoral and cellular immunity in pigs and poultry.

In summary, PRRs represent pivotal elements in the antiviral immune responses of livestock, with species-specific adaptations shaping their functional roles. Addressing the current gaps through multidisciplinary research will be key to developing next-generation antiviral therapies and vaccines tailored to the unique immunological landscapes of pigs and poultry. These efforts will not only enhance animal health and production efficiency but also contribute to broader goals in pandemic preparedness, food security, and zoonotic disease mitigation.
